# Targeting angiogenesis in endometriosis: a systematic review and network meta-analysis of VEGF-directed pharmacotherapies

**DOI:** 10.3389/frph.2025.1744465

**Published:** 2026-01-26

**Authors:** O. Hammond, O. El-Sheikh, R. M. Saad, P. Shetty, E. Papakonstantinou, S. L. Kastora

**Affiliations:** 1 University College Hospital, London, United Kingdom; 2 Chelsea and Westminster Hospital, London, United Kingdom; 3 Croydon Hospital, London, United Kingdom; 4 University College London, Womens’ Health Institute, London, United Kingdom; 5 Women’s Health Research Unit, Wolfson Institute of Population Health, Queen Mary University of London, London, United Kingdom

**Keywords:** endometriosis, fertility sparing, network analysis, systematic review, VEGF

## Abstract

**Background:**

Aberrant vascular endothelial growth factor (VEGF)–driven angiogenesis is central to the establishment and persistence of endometriosis. Although numerous anti-angiogenic compounds have been tested, evidence remains fragmented, and no comparative framework guides the selection of agents compatible with fertility preservation.

**Objective:**

To identify and rank vascular-targeted pharmacotherapies that most effectively regress endometriotic lesions and could be prioritised for fertility-sparing clinical translation.

**Methods:**

We conducted a systematic review and frequentist network meta-analysis (PROSPERO CRD420251082905) of controlled studies evaluating VEGF-directed agents in endometriosis. Six databases and two trial registries were searched from inception to 28 July 2025. Thirty-one studies met inclusion criteria (five early-phase human trials, six patient-derived cell models, two baboon experiments, and 18 rodent experiments) investigating 23 pharmacological agents. Primary outcomes were lesion area, lesion number, and VEGF expression; secondary outcomes included microvascular density, endometrial cell proliferation, and apoptosis. Random-effects pairwise and network models (R 4.3 “netmeta”) generated standardised mean differences (SMDs) with 95% CIs and SUCRA rankings. Transitivity, heterogeneity (*τ*^2^, *I*^2^), and inconsistency were formally assessed. Drug–gene target intersections across six cheminformatic databases mapped mechanistic convergence.

**Results:**

Curcumin achieved the greatest lesion-size reduction (SMD = −1.08, 95% CI = −1.38 to −0.79) and the steepest fall in microvascular density (−16%), while cetrorelix most effectively reduced lesion number (SMD = −0.78, 95% CI = −1.36 to −0.20). Retinoic acid and bevacizumab halved VEGF expression. Global inconsistency was non-significant (*P* > 0.18) and heterogeneity moderate (*I*^2^ ≤ 65%). A network analysis identified a 16-gene hub (*CASP3*, *MAPK1/3*, *AKT1*, *STAT3*, etc.) underpinning effective drugs; curcumin targeted 14 of these nodes.

**Limitations:**

Fifty-eight per cent of the data derived from rodent models revealed that these models do not menstruate and incompletely recapitulate human endometriosis. Most experiments reported short-term surrogate outcomes and rarely measured pain, fertility, or quality of life. Risk of bias was frequently moderate to high, and therefore, small-study or publication bias cannot be excluded.

**Conclusion:**

This study provides the first, exploratory comparative synthesis of VEGF-directed pharmacotherapies for endometriosis across pre-clinical and early-phase human models. Apparent efficacy rankings, including the high placement of curcumin, should be interpreted as relative signals within a limited and biased dataset rather than as evidence of inherent biological superiority or clinical readiness. These findings are best viewed as hypothesis-generating and may assist in prioritising agents for more rigorous translational and clinical research, rather than supporting specific treatment recommendations.

**Systematic Review Registration:**

PROSPERO CRD420251082905.

## Highlights

- The available evidence on VEGF-directed, potentially fertility-sparing treatments in endometriosis is sparse, short-term, and largely pre-clinical.
- Within this network of heterogeneous studies, several agents, including curcumin, cetrorelix, retinoic acid, and bevacizumab, show signals of lesion regression and VEGF pathway modulation.
- SUCRA rankings indicate relative performance within this dataset only and do not establish clinical efficacy or superiority; all findings should be considered only exploratory and hypothesis-generating.

## 1 Introduction

Endometriosis affects approximately one in ten women of reproductive age, eroding quality of life through chronic pelvic pain, dysmenorrhoea, and sub-fertility (1). Contemporary management still pivots on oestrogen-suppressive hormones or iterative surgery, interventions/approaches that can hinder fertility and are associated with recurrence rates of up to 50% within 5 years (2, 3). Mounting experimental evidence, however, highlights aberrant angiogenesis, primarily driven by vascular endothelial growth factor (VEGF), as central to lesion formation, innervation, and pain generation (4, 5). Targeting this vascular niche has therefore been proposed as a potential therapeutic strategy and is being explored in pre-clinical models as a route towards non-hormonal, fertility-compatible approaches.

As a result, a diverse pharmaco-armamentarium has emerged, including direct VEGF antibodies (6), multi-kinase inhibitors such as sorafenib; mTOR-PI3K modulators (rapamycin and temsirolimus); JNK inhibitors (bentamapimod); GnRH antagonists repurposed at angiostatic doses; dopamine agonists that down-regulate VEGF transcription; and pleiotropic nutraceuticals exemplified by curcumin (7, 8). However, these agents have been investigated in fragmentation, across disparate animal models and small pilot trials, leaving clinicians without a clear hierarchy of comparative effectiveness and researchers without a rational shortlist for translational prioritisation. Prior reviews are either narrative (5) or pairwise meta-analyses of single drug classes (62) and thus have been unable to meaningfully compare competing therapeutic options.

To date, VEGF-directed therapies in endometriosis remain pre-clinical, with only a very limited number of early-phase human studies that do not report on pain, fertility, or long-term safety. Against this backdrop, in this study, we undertake what is, to our knowledge, the first network meta-analysis (NMA) comparing all VEGF-targeted pharmacotherapies investigated in endometriosis, integrating animal and early-phase human data. Utilising contemporary cheminformatic resources (9), we layer a pathway-enrichment screen onto the quantitative synthesis, aiming to expose mechanistic convergence and nominate additional candidates for disease-modifying, conception-compatible treatment. Nonetheless, the present work is not intended to provide clinical recommendations. Instead, our aim is to synthesise the diverse pre-clinical and early-phase evidence on VEGF-directed pharmacotherapies to describe patterns of relative efficacy on lesion-level outcomes across models and generate hypotheses that may inform future mechanistic and translational research.

## 2 Methods

### 2.1 Search strategy and information sources

A comprehensive electronic search, prospectively registered in PROSPERO (CRD420251082905), was executed on 31 October 2024 and updated on 28 July 2025, from database inception, across MEDLINE (OVID-SP), Embase, Scopus, Cochrane CENTRAL, Google Scholar, and ClinicalTrials.gov. Two research registries (MedRxiv and Research Square) were also enquired. The core string combined controlled vocabulary and free-text synonyms: (“vascular endothelial growth factor” OR VEGF OR Angiogenesis) AND endometriosis AND (treatment OR management OR therap*), with field limits set to Title/Abstract. No restrictions were imposed regarding language, organism, or publication date. Reference lists of relevant systematic reviews and eligible meta-analyses were hand-searched for additional studies (Supplementary Figure S1).

### 2.2 Eligibility criteria

Randomised and non-randomised controlled trials, comparative cohort studies, and controlled laboratory experiments (murine or primate) that evaluated a systemic or local pharmacological agent acting on VEGF signalling in surgically or histologically confirmed endometriosis were included. We defined “VEGF-directed” pharmacotherapies *a priori* as systemic or local agents with a primary or well-described secondary mechanism involving VEGF signalling or angiogenesis modulation (anti-VEGF antibodies, multi-kinase inhibitors targeting VEGFR signalling, mTOR-PI3K modulators, GnRH analogues/dopamine agonists with documented anti-angiogenic effects, and pleiotropic compounds with reproducible VEGF down-regulation in experimental models). We required studies to report at least one lesion-level outcome (lesion area, lesion number, or VEGF expression within ectopic tissue). Studies limited to in vitro assays without a lesion-bearing model, narrative reviews, and non-comparative series were excluded. Narrative reviews, *in vitro* only studies, conference abstracts without full text, and papers lacking extractable outcome data were excluded. Three reviewers (SLK, OH, and PS) independently screened titles and abstracts, followed by a full-text review. An independent fourth reviewer (OE) assessed the final set of texts included in the study (Supplementary Figure S1).

### 2.3 Objectives

The present study aimed to quantify the effectiveness of fertility-sparing treatments, outside of the hormone suppression blanket' across clinical and histological parameters relevant to disease control. In addition, the level of evidence certainty, associated heterogeneity, and organism-level divergence was assessed.

### 2.4 Outcomes

Primary outcomes were defined as (i) lesion area, measured in mm^2^ or as a volumetric surrogate and extracted as mean change from baseline or between-group difference at endpoint; (ii) lesion number, expressed as absolute count or percentage reduction; and (iii) VEGF expression within ectopic lesions, assessed by immunohistochemistry, ELISA, or mRNA quantification and normalised to control tissue when possible. Secondary outcomes were microvascular density (MVD), endometrial cell proliferation (Ki-67 markers), and apoptosis (TUNEL, caspase-3). Outcomes were normalised where feasible as ratios of (post-control − post-treatment)/post-control to facilitate comparison across models.

### 2.5 Data extraction

Using a piloted extraction sheet (Excel 2023), SLK and OH captured study design, population characteristics, intervention details (agent, dose, route, and duration), comparator, and outcome data [mean ± SD or median (IQR)] (Supplementary Table S1). Reasons for exclusion at the eligibility stage were documented. Data accuracy was confirmed by two reviewers (RMS and EP), who performed a 20% random audit for transcription accuracy; no discrepancies > 3% were detected.

### 2.6 Risk-of-bias appraisal and certainty in evidence

Observational or quasi-randomised studies trials (*N* = 3) were assessed using the ROBINS-I tool (Supplementary Table S2) and animal studies using SYRCLE's checklist (Supplementary Table S3). Judgements were made independently by SLK and OH; consensus was sought through discussion or, if needed, through arbitration by OKE (Supplementary Figure S2). The GRADE framework was adapted for the network meta-analysis, downgrading for risk of bias, indirectness, inconsistency, imprecision, and publication bias. Summary of findings tables was generated with *CINeMA*.

### 2.7 Statistical synthesis

Where ≥2 studies reported a common outcome, pairwise meta-analyses were performed in R 4.3.2 (*meta* and *netmeta* packages) using inverse-variance random-effects models (Hartung–Knapp adjustment). For network geometry with three or more competing interventions, a frequentist random-effects network meta-analysis was performed, estimating pooled relative effects as standardised mean differences and 95% confidence intervals (continuous outcomes). Surface under the cumulative ranking curve (SUCRA) values were calculated for each agent to summarise the probability of that treatment being among the best within the observed network. Notably, SUCRA values should be interpreted as relative rankings conditional on the included studies and network structure and not as absolute measures of biological efficacy or clinical usefulness. Transitivity was examined clinically and statistically; global inconsistency was probed using the design-by-treatment interaction model and local inconsistency using node splitting (Supplementary Figure S3). Heterogeneity was quantified by *τ*^2^ and *I*^2^, and publication bias was examined using comparison-adjusted funnel plots where ≥10 trials contributed. Pre-specified sensitivity analyses included (i) restriction to non-rodent (“human/humanoid”: human, patient-derived cells, and primates) models and (ii) exclusion of single-study nodes. These were used to assess the robustness of treatment rankings to species and network sparsity.

### 2.8 Drug–gene target mapping

The target-mapping workflow was initiated by harmonising all chemical–gene associations of Homo sapiens for each analysed index compound, drawing from the Comparative Toxicogenomics Database (9), STITCH v5.0 (10), the DrugBank open subset (11), ChEMBL v33 (12), and the complementary patent- and GWAS-driven resources DGIdb v5 (13) and Open Targets 23.12 (14). The most potent measurement per drug–gene dyad was retained and duplicate HGNC symbols were collapsed into a single non-redundant list. The resultant sets were imported into a Python 3.13/Miniforge environment, intersected by straightforward set algebra and rendered through the UpSet algorithm (15), with a putative human background of 20,000 protein-coding genes. Genes present in at least two drugs (*N* = 37, Supplementary Table S2) were interrogated for functional over-representation against KEGG (16), Reactome (17), and WikiPathways (18) via clusterProfiler 4.10 (19) and cross-validated in g:Profiler2 (20), adopting a Benjamini–Hochberg FDR <0.05 (Supplementary Table S4). The analysis converged on a 16-gene kernel encompassing caspase-mediated apoptosis, MAPK/VEGF-downstream signalling, p53 control, and a sirtuin node (Figure 3, Supplementary Table S5).

### 2.9 Exploration of untested drug combinations

To prioritise fertility-sparing candidates, we mapped every drug-specific target list onto the KEGG and Reactome pathways that emerged as significantly enriched in the common-gene analysis (Supplementary Table S2). Gene–drug matrices were constructed in Python 3.13 (pandas 2.2) and interrogated using the GSEApy wrapper for clusterProfiler 4.10 and ReactomePA. In enrichment statistics, we used a one-tailed hyper-geometric test against the 20,000-gene human universe with Benjamini–Hochberg correction (*α* = 0.05). For each compound, we calculated a “pathway-coverage score”—the proportion of hall-mark genes (*N* = 16) captured within the apoptosis/caspase, MAPK, PI3K-Akt/mTOR, and VEGF modules (STRINGApp, edge-confidence ≥ 0.7). The drugs were then ranked by coverage score, after which safety descriptors (pregnancy registry data and EMA/FDA labels) were overlaid in R 4.3 using dplyr and ggplot2 to flag regimens compatible with conception (Supplementary Table S6).

## 3 Results

### 3.1 Included study characteristics and risk of bias

**Figure 1:**
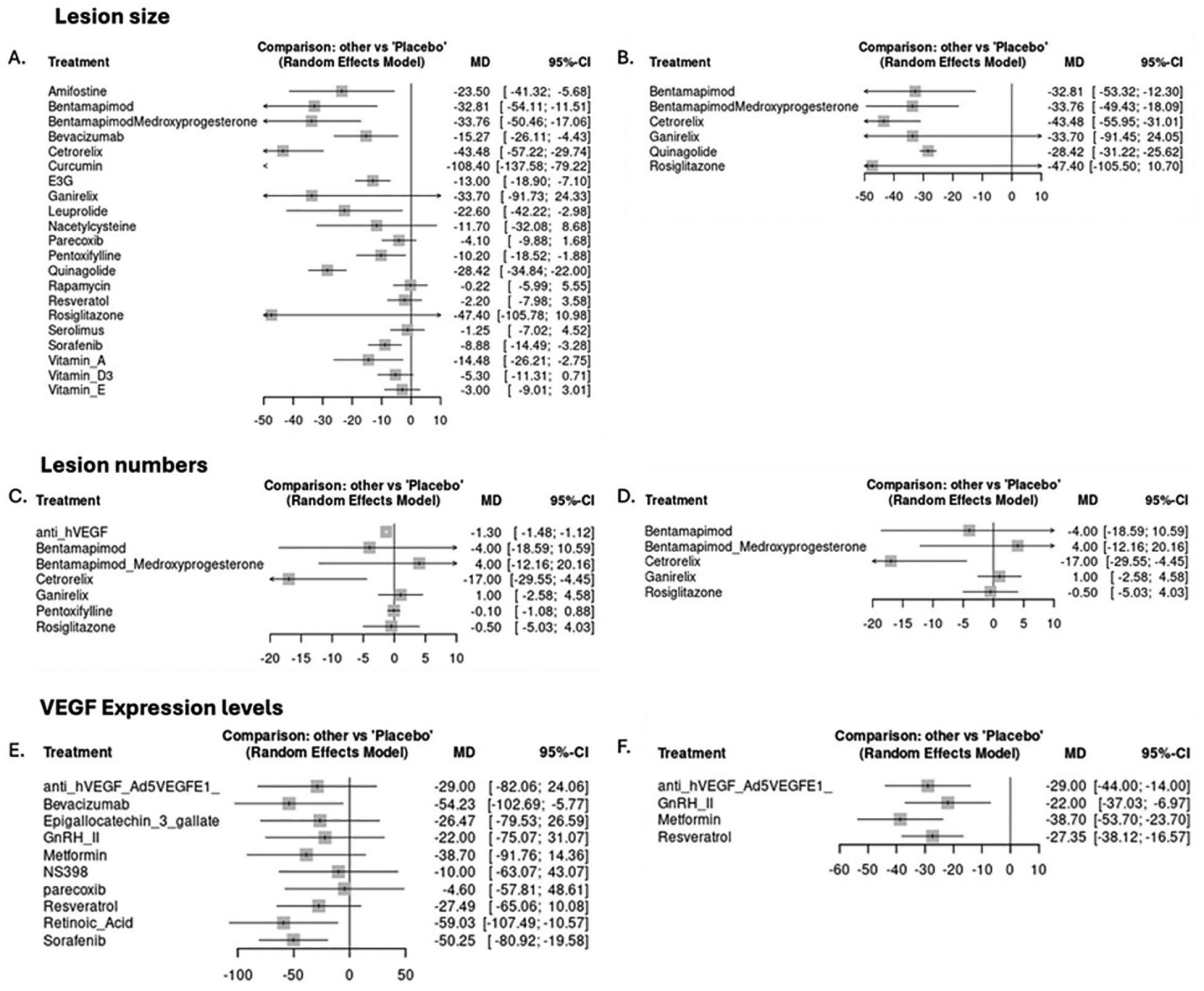
Primary outcomes: lesion size in mm^2^, lesion numbers MD of post-placebo-post-treatment/post-placebo, and VEGF expression difference MD of post-placebo-post-treatment/post-placebo. Left graphs: human; human cell lines; primates; rodents. Right graphs: human; human cell lines; primates.

**Figure 2:**
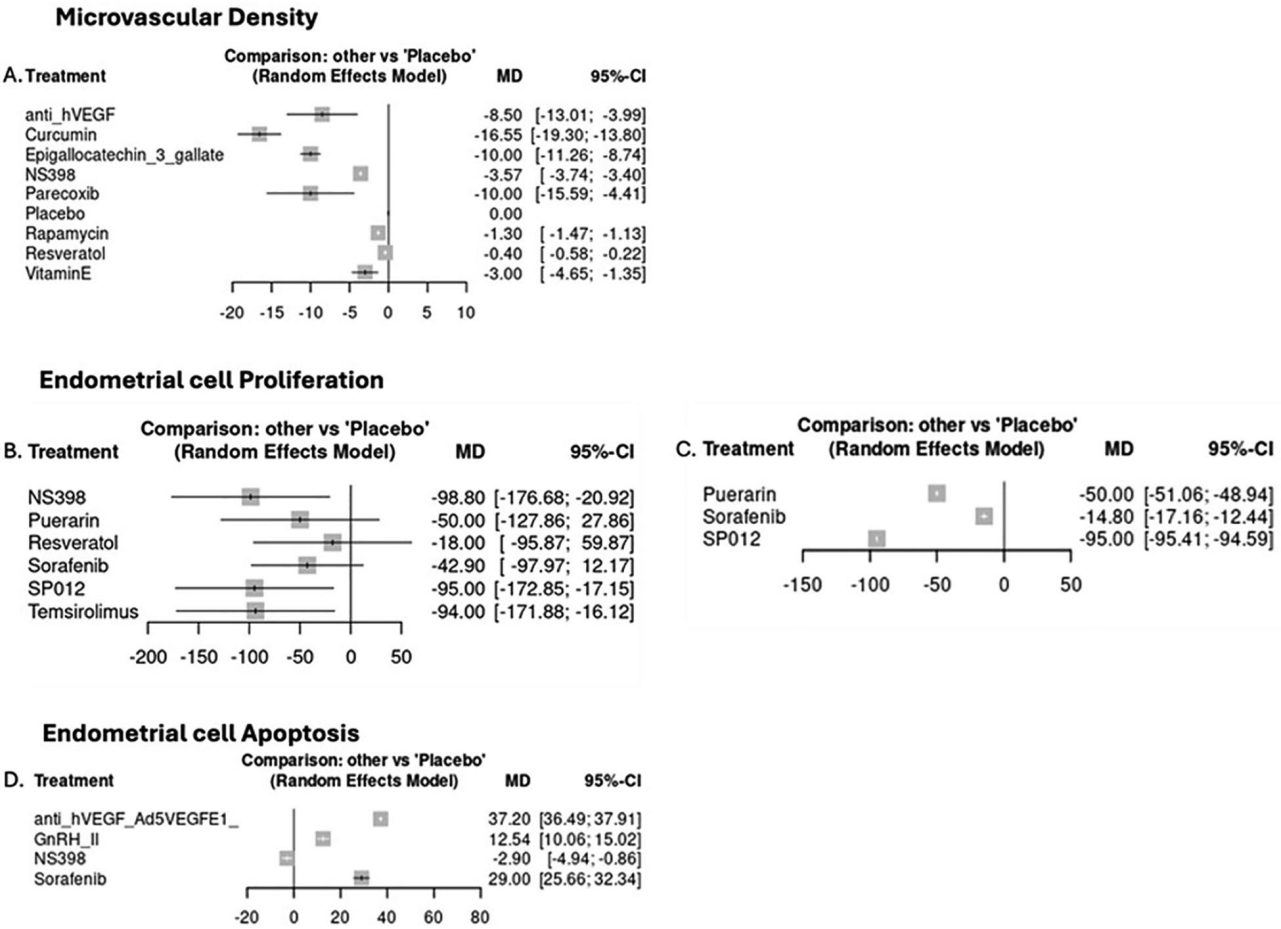
Secondary outcomes: microvascular density reduction in % post-placebo-post-treatment/post-placebo, proliferation increase/reduction in % post-placebo-post-treatment/post-placebo, and apoptosis increase/reduction in % post-placebo-post-treatment/post-placebo. Left graphs: human; human cell lines; primates; rodents. Right graphs: human; human cell lines; primates (where ≥2 studies reporting).

A total of 31 studies, six conducted in humans (21–26), six *in vitro* studies with patient isolated cells (27–32), two conducted in baboons (33, 34), and 17 performed in rodents (4, 35–47, 63, 64) met the eligibility criteria and contributed to the network meta-analysis (Supplementary Figure S1, Supplementary Table S1). Across all models, the primary outcomes were expressed as mean difference (MD) in absolute lesion area (mm^2^), lesion number reduction (%), VEGF expression reduction (%), vs. placebo. The secondary outcomes were expressed as microvessel density, apoptosis, and proliferation reduction (−%) or increase (−%). The results were reported first for the complete network (“all organisms,” Figures 1A,C,E, 2A,B,D) and then for the restricted network that excluded rodent data to probe inter-species robustness (Figures 1B,D,F, 2C). Risk of bias was assessed in accordance with human-based studies through the Robins-I framework (Supplementary Table S2), with an overall moderate risk of bias, and in accordance with an animal-based study using the SYRCLE checklist, with an overall fair quality of included studies (Supplementary Table S4). Pooled SUCRA values placed bentamapimod, ganirelix, cetrorelix, curcumin, and sorafenib in the top quintile of efficacy, whereas antioxidant vitamins and non-selective NSAIDs clustered near placebo (Supplementary Figure S4); individual study results were analysed to correlate study-level outcomes (direct) with NMA-level cumulative (mixed) outcomes (Supplementary Figure S5).

### 3.2 Overall league of interventions and sensitivity analysis excluding rodent organisms

#### 3.2.1 Primary outcomes: lesion number, size, and VEGF expression levels

Within the combined network of pre-clinical and early-phase studies, curcumin was associated with the largest pooled reduction in lesion size (MD = −108.40 mm^2^; 95% CI = −137.58 to −79.22), followed by rosiglitazone (MD = −47.4 mm^2^; 95% CI = −105.78 to 10.98), ganirelix (−33.7 mm^2^; −41.3 to −26.1), and bentamapimod (MD = −33.2 mm^2^; 95% CI = −54.3 to −12.1 (Figure 1A). The effect was maintained in only the “human/humanoid” organism subgroup analysis, with rosiglitazone achieving a 47% reduction in lesion size (Figure 1B). Cetrorelix was the most effective treatment in reducing lesion numbers (MD = −17.0; 95% CI = −29.55 to −4.45), an effect that was maintained in the subgroup analysis (Figures 1C,D). Retinoic acid (MD = −59.03; 95% CI = −107.49 to −10.57) and bevacizumab (MD = −54.23; 95% CI = −102.69 to −5.77) achieved an over 50% reduction in VEGF expression levels, followed by metformin (MD = −38.70; 95% CI = −91.76 to 14.39) (Figure 1E). The metformin effect was maintained in the subgroup analysis (MD = −38.70; 95% CI = −53.70 to 23.70) (Figure 1F). According to outcomes such as lesion number, lesion size, and VEGF expression levels, heterogeneity was moderate (*I*^2^ = 47%, *I*^2^ = 65%, *I*^2^ = 54%) and the design-by-treatment test detected no global inconsistency (*P* = 0.21, *P* = 0.18, *P* = 0.14). SUCRA values suggested that bentamapimod, ganirelix, cetrorelix, curcumin, and sorafenib ranked in the upper range of relative performance; however, confidence intervals for many pairwise contrasts were wide and overlapping, and rankings should therefore be interpreted as probabilistic summaries rather than evidence of intrinsic superiority. We did not detect statistically significant global or local inconsistency in either the full or non-rodent networks (design-by-treatment *P* = 0.34, *P* = 0.12, *P* = 0.30 respectively), but given the small number of studies per comparison, these tests are underpowered and cannot exclude clinically relevant discrepancies between species.

#### 3.2.2 Secondary outcomes: microvascular density, endometrial cell proliferation, and apoptosis

Curcumin achieved the most notable reduction in MVD (MD = −16.55; 95% CI = −19.30 to −13.80), followed by epigallocatechin-3-gallate (E3G) (MD = −10.0; 95% CI = −11.26 to −8.74) and parecoxib (MD = −10.0; 95% CI = −15.59 to −4.41) (Figure 2A). All studies reporting MVD were conducted on rodent models; therefore, a subsequent subgroup analysis was not feasible. With regard to endometrial cell proliferation (Ki67 expression), NS398, SP012, and temsirolimus achieved comparable control upon cellular proliferation (MD = −98%, −95%, and −94%) respectively, with the SP012 effect confirmed in non-rodent models (MD = −95; 95% CI = −95.41 to −94.59) (Figures 2B,C); anti-VEGF antibody (MD = 37.20, 95% CI = 36.49 to 37.91) and sorafenib (MD = 29, 95% CI = −25.66 to 32.44). According to the outcome, heterogeneity was low (*I*^2^ = 12%, *I*^2^ = 52%, *I*^2^ = 20%) and the design-by-treatment test detected no global inconsistency (*P* = 0.28, *P* = 0.35, *P* = 0.15). Similar to the primary outcome analysis, network inconsistency remained non-significant in the human/humanoid subgroup analysis for endometrial cell proliferation (*P* = 0.60). Overall, comparison-adjusted funnel plots were generated for outcomes with ≥10 contributing studies (Supplementary Figures S3, S4). A visual inspection suggested some asymmetry, consistent with a possible small-study or publication bias, although the limited number of studies in most contrasts precludes firm conclusions (Supplementary Figure S6).

### 3.3 Molecular convergence of effective agents

**Figure 3:**
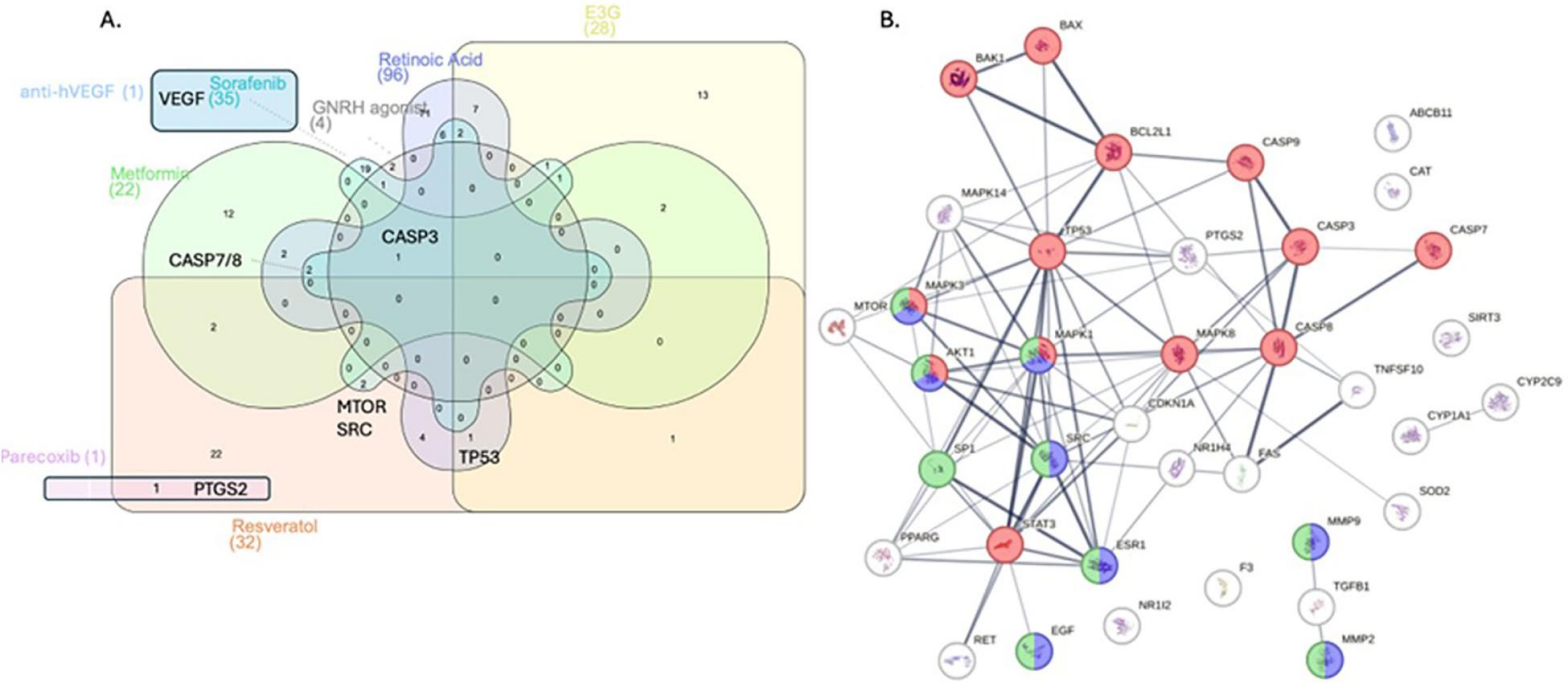
An overlap of the genetic targets of NMA-identified drugs with statistically significant impact on two-third of primary outcomes and one-third of secondary outcomes. Red nodes: intrinsic apoptosis pathway; Blue: extra-nuclear aestrogen signalling; Green: ESR-mediated signalling.

In an exploratory *in silico* analysis based on curated drug–gene interaction databases, the Venn–STRING composite suggested a star-shaped overlap centred on CASP3, with 22–36 peripheral genes per drug (Figures 3A,B, Supplementary Tables S4, S5). Bentamapimod, curcumin, and sorafenib shared targets in MAPK1/3, RAF1, and AKT1, which is consistent with their observed anti-proliferative signals *in vivo*. Ganirelix and cetrorelix contributed a GNRHR–CASP3–MAPK1 sub-module, while anti-VEGF antibodies selectively docked VEGFA. Pathway enrichment mapped these intersections to MAPK signalling, PI3K-Akt/mTOR, apoptotic caspases, and VEGF-driven angiogenesis. These findings should be interpreted as hypothesis-generating rather than as mechanistic proof.

Among agents with lesion regression signals, curcumin and quinagolide had the broadest coverage of the 16-gene hub without direct mTOR inhibition or systemic endocrine suppression (Figure 3A, Supplementary Table S5). Tacrolimus, when modelled alongside curcumin in this framework, theoretically expanded pathway coverage, while avoiding some of the gonadotoxicity associated with sirolimus. We further compared fertility-sparing vs. non-sparing agents against the drug–gene overlapping pathway to detect putatively clinically meaningful, pharmaceutical targets (Supplementary Table S6). With regard to fertility-sparing agents, curcumin ranked first (pathway coverage 87%), while sorafenib ranked first from the fertility non-sparing agents (pathway coverage 38%). These patterns are purely *in silico* and were presented only to prioritise combinations for further pre-clinical evaluation. Therefore, they do not constitute evidence that such regimens would be effective or safe in humans.

## 4 Discussion

The present systematic review and network meta-analysis integrated 31 controlled studies of 23 vascular-targeted agents across rodents, baboons, patient-derived stromal cultures, and a small number of early-phase human trials. Several classes of VEGF-modulating therapies, including anti-VEGF antibodies, GnRH antagonists, multi-kinase inhibitors, mTOR-pathway modulators, and pleiotropic nutraceuticals, showed signals of reduced lesion size, lesion number, or VEGF expression. However, these signals rose predominantly from short-term, heterogeneous rodent models and must therefore be interpreted as preliminary and hypothesis-generating.

Within this pre-clinical network, curcumin consistently occupied a high position in SUCRA-based rankings for lesion size and microvascular density, linked to a broad multi-target profile in exploratory drug–gene analyses. It is important, however, not to over-interpret this apparent “dominance.” First, curcumin's effect estimates derive almost entirely from small rodent experiments, with considerable variability in formulation, dosing, and outcome measurement. Second, curcumin is well known to have poor and formulation-dependent oral bioavailability, raising questions about whether the exposures achieved in animal models are attainable or relevant in humans. Third, human data for curcumin in endometriosis are essentially not available. Therefore, at this stage, the high ranking of curcumin in our NMA should be regarded as a statistical artefact of the available dataset caused by a signal that this compound has attracted a more intensive pre-clinical study, rather than an evidence of intrinsically superior therapeutic potential or clinical readiness.

In recent years, it has become apparent that the therapeutic landscape of endometriosis is not one-size-fits-all. Women with advanced, painful disease who have completed their family wishes or are facing imminent surgical menopause, require lesion eradication rather than fertility preservation. The present analysis has suggested that “potency-first” experimental agents such as sorafenib, sirolimus/everolimus, and bentamapimod regress lesions by 30%–50%, magnitudes rarely attained with oestrogen suppression (5, 34). These agents lie outside the conception-compatible window as they interfere with folliculogenesis, placental VEGF signalling, or embryo JNK-MAPK homeostasis. However, these agents can prove valuable for refractory pelvic pain, deep infiltrating disease that threatens adjacent organs, or interval cytoreduction before definitive surgery. In this context, hormonal agents such as the combined contraceptive pill, progestins, and GnRH analogue suppression remain the gold standard for endometriosis medical management, for disease control till surgery, and for post-surgical maintenance of disease regression (48).

However, despite the longstanding clinical narrative that “pregnancy cures endometriosis,” modern evidence offers a more nuanced perspective. Prospective ultrasound studies have shown that approximately 85% of endometriomas and nodules shrink during gestation, yet only 15% disappear completely, with the majority recurring after delivery (49). Furthermore, nearly all licensed medical therapies rely on ovulation or systemic oestrogen suppression, effectively excluding patients actively pursuing conception and leaving a significant gap for truly fertility-sparing, disease-modifying drugs (50). Therefore, patients desiring pregnancy, require anti-angiogenic regimens that dismantle lesions without obstructing ovulation and/or implantation or harbor teratogenic potential. However, studies exploring such options remain scarce. In this context, the present analysis suggests that curcumin fulfils this brief on three fronts. First, it possesses unparalleled pathway breadth, striking 14/16 genes in the endometriosis “hub,” while avoiding gonadotoxic mTOR inhibition (51). Second, curcumins’ safety record spans culinary exposure to oncology phase II trials in which oral doses up to 8 g day^−1^ cause mild gastrointestinal symptoms (52). Third, pharmacodynamic modelling suggests that 1 g day^−1^ of a nanoparticle or phospholipid complex reaches plasma levels sufficient to inhibit VEGF, MAPK, and STAT3 signalling, whilst dose escalation to 2 g day^−1^ can be achievable. However, despite the putative mechanistic benefit of curcumin, further pre-clinical studies are direly required to advise on dose standardisation, subjective effect measurement, and applicability in clinical-level studies (55–61).

### 4.1 Future research perspectives

Combinatorial therapy appears a promising option, although no current studies have explored this therapeutic avenue. Add-on low-exposure tacrolimus would supply NFAT-cytokine blockade that curcumin lacks, covering the remaining two untargeted genes in the hub without imposing teratogenic potential (53, 54). Alternatively, quinagolide may present a second, endocrine-neutral partner that down-regulates VEGF transcription via dopaminergic signalling. Importantly, future studies should build on the observations of the present analysis in a staged manner. Initial work will be required to optimise dosing, formulation, and delivery of candidate agents such as curcumin and also in relevant animal models, including a rigorous assessment of reproductive and embryonic safety. Only after reproducible lesion-level and mechanistic effects are demonstrated in multiple species should early-phase human trials be contemplated. If such trials are pursued, they should prioritise patient-centred outcomes such as pain, quality of life, and fertility as well as long-term safety, rather than relying solely on anatomical endpoints.

### 4.2 Limitations

This synthesis is constrained by the biological and methodological limitations of the underlying evidence. Fifty-eight per cent of the pooled data were derived from rodent models, which do not recapitulate human stromal–immune crosstalk and typically rely on ectopic transplantation under high-oestrogen conditions. Resultant lesions evolve over weeks rather than years and may not capture the chronic inflammatory, fibrotic, and neuropathic features of human disease. Even among non-rodent and human-derived models, there was substantial heterogeneity in lesion induction, dosing schedules, and outcome definitions. Most experiments were short term (<12 weeks) and focused on surrogate anatomical endpoints, with almost no data on nociceptive behaviour, quality of life, fertility, or long-term safety. These species- and experiment-level constraints mean that our effect estimates should be viewed as preliminary indicators of biological activity rather than as predictors of clinical benefit. Risk-of-bias appraisal was frequently non-trivial, with only about one-third of animal studies clearly reporting randomisation, allocation concealment, or blinded outcome assessment, and non-randomised human cohorts were prone to residual confounding, including by disease stage. Several network nodes were informed by few and often small studies, yielding wide and overlapping confidence intervals. In this context, the SUCRA-based hierarchy reflected relative rankings within a sparse and heterogeneous dataset and should be interpreted as a heuristic to prioritise candidates for further investigation and not as evidence of inherent biological superiority or clinical precision. Comparison-adjusted funnel plots were compatible with possible small-study and publication bias, particularly for lesser-studied agents, although the limited number of trials per comparison undermined formal detection, and dose–response data were too sparse to support modelling of optimal human dosing. Finally, our drug–gene intersection and pathway-enrichment analyses were strictly in silico overlays that depended on incomplete, curated interaction databases. Therefore, they could not be used to establish causality nor to confirm target engagement at clinically achievable exposures, and their role is limited to prioritising mechanistic hypotheses for future pre-clinical and translational work.

Despite these limitations, our meta-network integrated animal, organoid, and first-in-human data into a hypothesis-driven, efficacy analysis yet for anti-angiogenic drugs in endometriosis treatment. Rigorous duplicate screening, full bias scoring, and design-by-treatment tests supported the identification of putative therapeutic targets. Furthermore, the present NMA classified higher-ranking compounds into drug–gene maps to explore biologically relevant and clinically plausible recommendations for future research.

## 5 Conclusion

Endometriotic lesions depend on aberrant angiogenesis for their growth and persistence, and our synthesis suggests that several VEGF-modulating strategies can disrupt this process in pre-clinical models. However, the underlying evidence is short term, heterogeneous, and heavily weighted towards rodent experiments with important translational limitations. Our network meta-analysis and exploratory bioinformatic overlay should therefore be understood as a hypothesis-generating exercise that highlights candidate agents and pathways. Future research should prioritise rigorous, transparently reported pre-clinical studies that incorporate pain and fertility endpoints, followed by carefully phased clinical trials, before any VEGF-directed therapy can be considered for routine use in women with endometriosis.

## Data Availability

The original contributions presented in the study are included in the article/Supplementary Material, and further inquiries can be directed to the corresponding author.

## Supplementary material

Supplementary Figure S1 PRISMA Chart.

Supplementary Figure S2 Risk of Bias assessment of included studies.

Supplementary Figure S3 Networks per outcome.

Supplementary Figure S4 SUCRA Ranking plots.

Supplementary Figure S5 Individual study results (all included studies) grouped by treatment comparison.

Supplementary Figure S6 Comparison-Adjusted Funnel Plots (CAFP) per outcome. Precision against comparison adjusted effect vs placebo for the following outcomes: Lesion number, lesion size, VEGF expression, Proliferation, Apoptosis and Microvascular density.
