# Supplementary figures and images for: Targeting angiogenesis in endometriosis: a systematic review and network meta-analysis of VEGF-directed pharmacotherapies
Source: Front Reprod Health. 2026 Jan 26;7:1744465. doi: 10.3389/frph.2025.1744465 (PMC12883779; doi:10.3389/frph.2025.1744465)

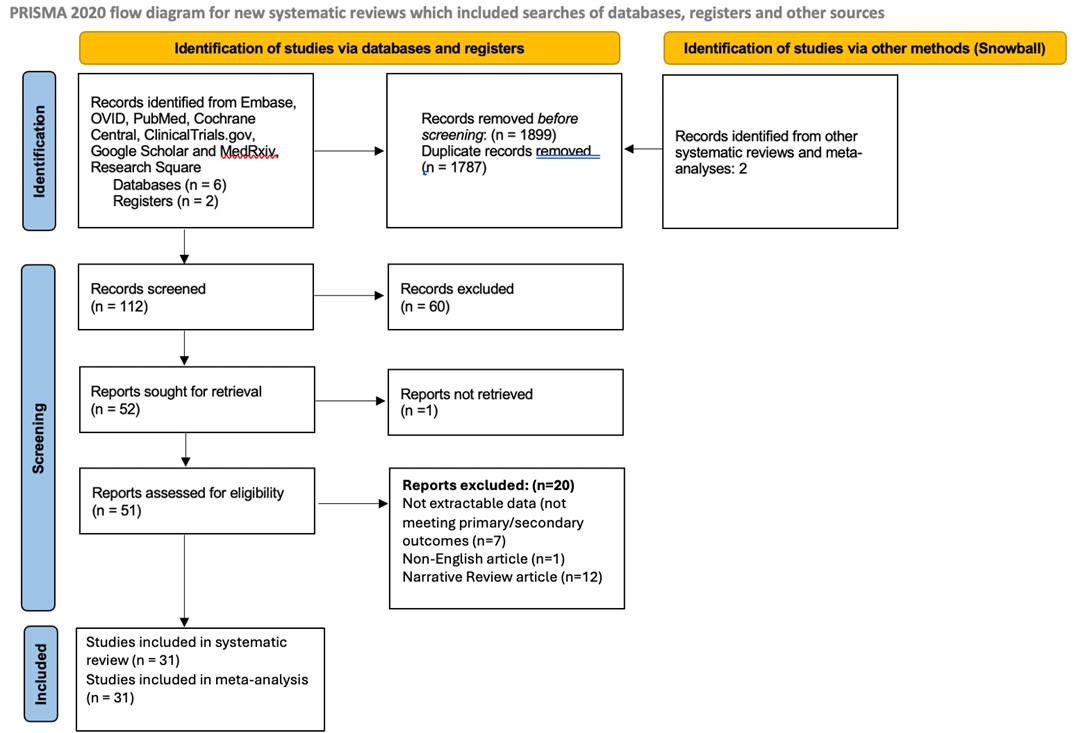

Supplement: Supplementary Figure S1 — PRISMA Chart. [file Figure1.jpg]

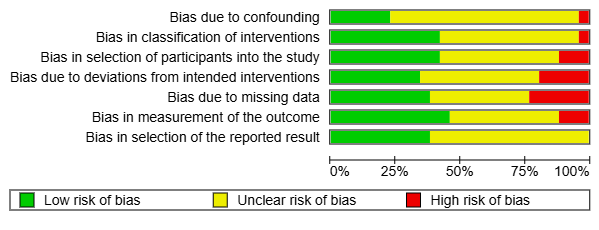

Supplement: Supplementary Figure S2 — Risk of Bias assessment of included studies. [file Figure2.png]

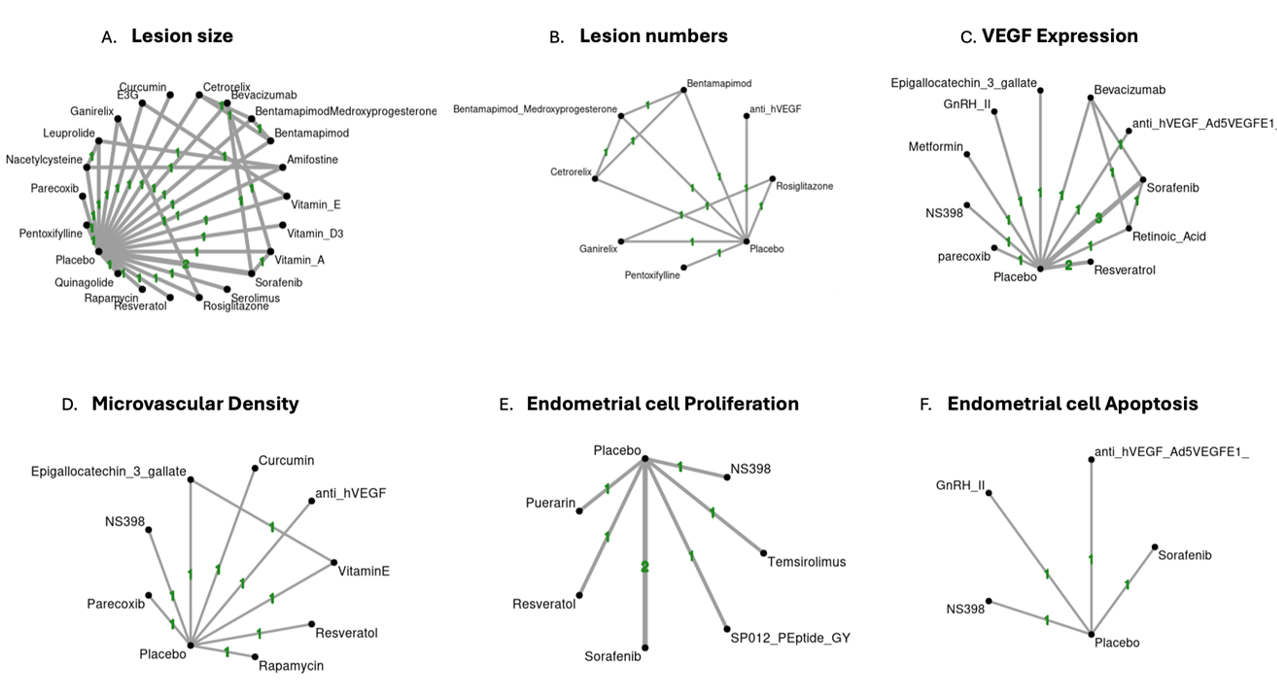

Supplement: Supplementary Figure S3 — Networks per outcome. [file Figure3.png]

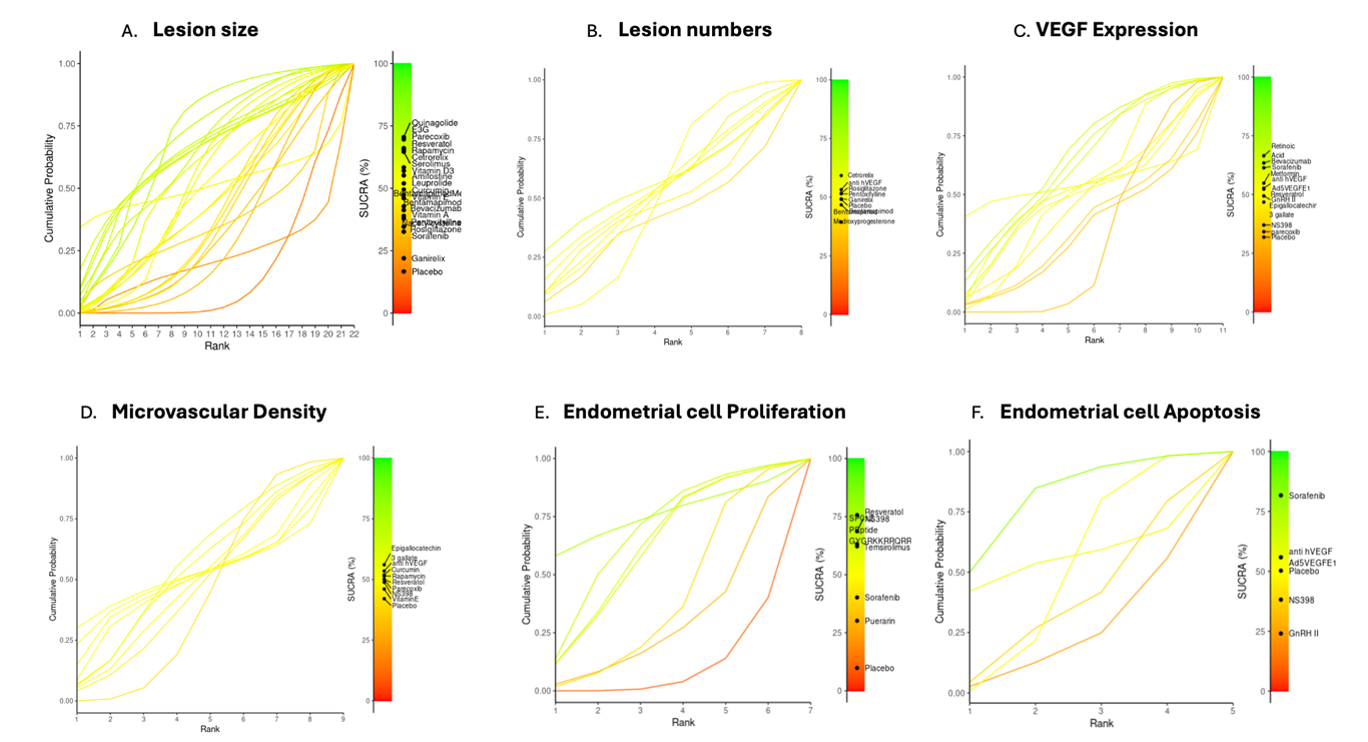

Supplement: Supplementary Figure S4 — SUCRA Ranking plots. [file Figure4.png]

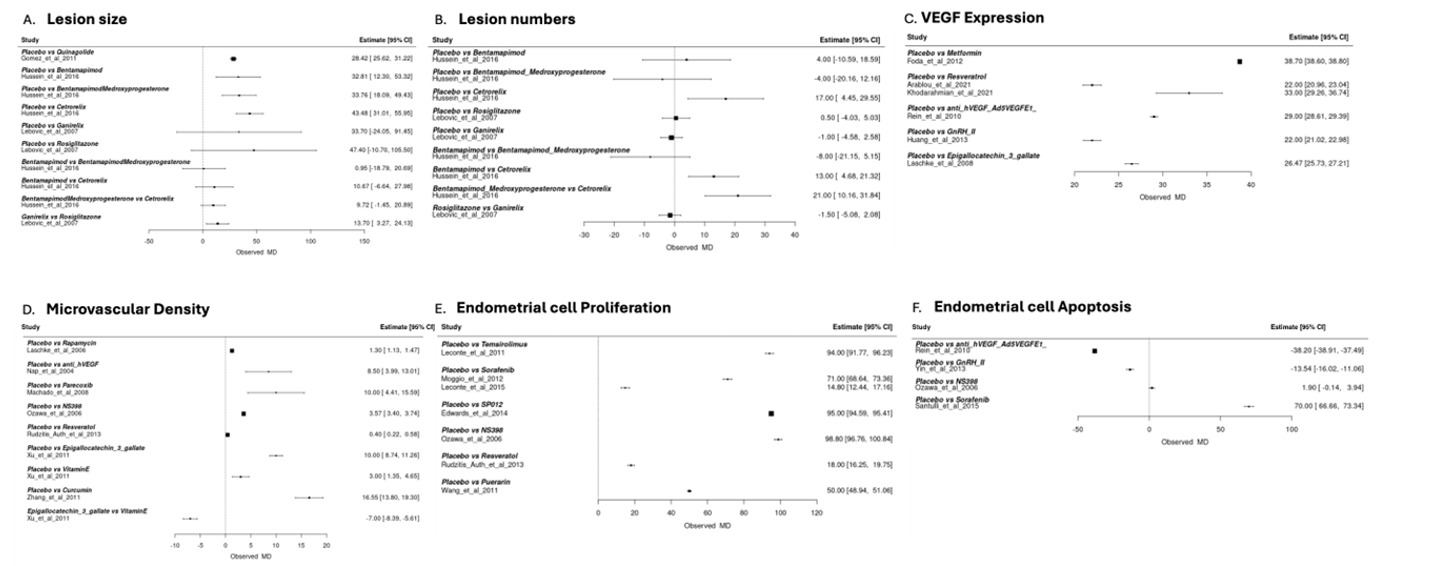

Supplement: Supplementary Figure S5 — Individual study results (all included studies) grouped by treatment comparison. [file Figure5.png]

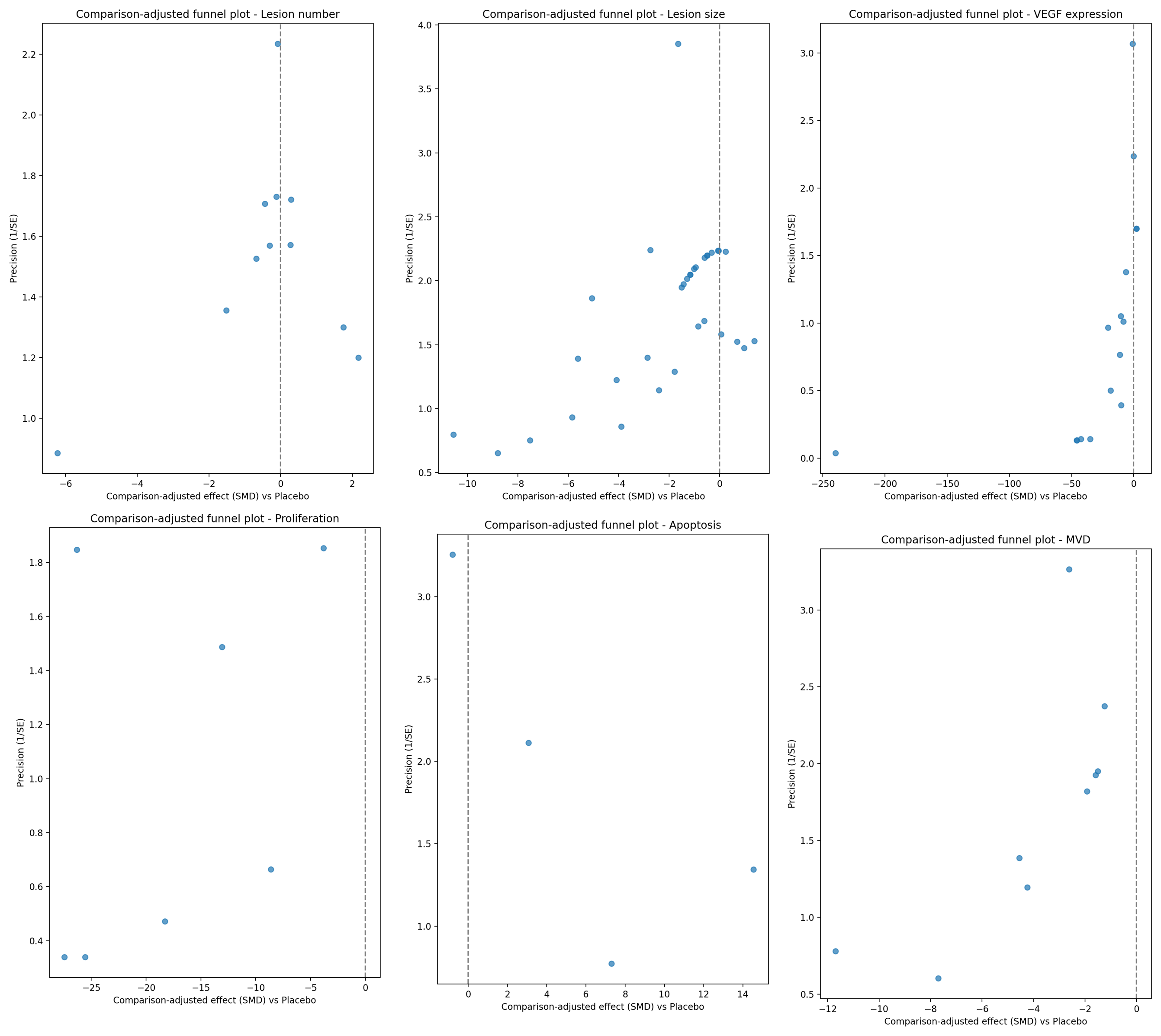

Supplement: Supplementary Figure S6 — Comparison-Adjusted Funnel Plots (CAFP) per outcome. Precision against comparison adjusted effect vs placebo for the following outcomes: Lesion number, lesion size, VEGF expression, Proliferation, Apoptosis and Microvascular density. [file Figure6.png]
