# Supplementary material for: Targeting angiogenesis in endometriosis: a systematic review and network meta-analysis of VEGF-directed pharmacotherapies
Source: Front Reprod Health. 2026 Jan 26;7:1744465. doi: 10.3389/frph.2025.1744465 (PMC12883779; doi:10.3389/frph.2025.1744465)
Supplement: Supplementary file 7 [file Supplementaryfile1.docx]

Title. Targeting Angiogenesis in Endometriosis: A Systematic Review and Network Meta-analysis of VEGF-Directed Pharmacotherapies.

**Authors** : Hammond O.1, El-Sheikh O.1, Saad RM.2, Shetty P.1, Papakonstantinou E.3 Kastora SL.4

| **Study** | **Type of Study** | **Year of study** | **Organism Tested** | **Compound tested** | **Exposed cohort [N]** | **Control cohort [N]** |
| --- | --- | --- | --- | --- | --- | --- |
| Arablou et al., 2021 | In vitro study | 2021 | Human endometrial stromal cells (ESCs) | Resveratrol | 32 | 32 |
| Foda et al., 2012 | Prospective study | 2012 | Humans | Metformin | 20 | 20 |
| Goncalves et al., 2014 | In vitro animal study | 2015 | Human endometrial stromal cells | p27^kip1 (via adenoviral vector) | Not specified | Not specified |
| Filindris et al., 2024 | Prospective study | 2024 | Humans | Leuprolide acetate (GnRH agonist) | 30 | 30 |
| Leconte et al., 2015 | In vitro and in vivo animal study | 2015 | Human endometrial stromal cells and mouse models | Sorafenib (multikinase inhibitor targeting RAF kinase and VEGFR) | 10 | 10 |
| Moggio et al., 2012 | In vitro study | 2012 | Human | Sorafenib | 6 | 6 |
| Nap et al., 2004 | In vitro and in vivo experimental study | 2004 | Human/Mouse | anti-hVEGF | 10 | 10 |
| Machado et al., 2008 | Case control | 2008 | Female Sprague-Dawley rats | Parecoxib | 10 | 10 |
| Okada et al., 2001 | In vitro study | 2001 | Human | Oestradiol (10–8 mol/l) plus dienogest, RU-486 | 3 | 3 |
| Gomez et al., 2011 | In vitro and in vivo experimental study | 2011 | Human | Dopamine agonists (quinagolide) | 9 | 0 |
| Rein et al., 2010 | Case control | 2010 | Human / mouse | VEGF-targeted conditionally replicative adenovirus (Ad5VEGFE1) | 60 | 3 |
| Santulli et al., 2015 | Randomised control trial | 2015 | Mouse - endometrial samples | Sorafenib | 15 | 15 |
| Khodarahmian et al., 2021 | Randomised control trial | 2021 | Human | Resveratrol | 17 | 17 |
| Huang et al., 2013 | Case control | 2013 | Human/ESC | GnRH-II | 30 | 16 |
| Abbas et al, 2013 | Case control | 2013 | Rats | Vitamin D3 | 9 | 8 |
| Edwards et al, 2014 | In vivo and in vitro | 2014 | Human and mouse | SP012 (12 amino acid peptide inhibitor of synuclein-γ) | 4 | 4 |
| Hussein et al, 2016 | Prospective randomized study | 2016 | Baboons | Bentamapimod | 3 | 5 |
| Laschke et al, 2006 | In vivo animal study | 2006 | Mouse | VEGF-R2 tyrosine kinase inhibitor, PTK787/ZK222584 | 10 | 10 |
| Laschke et al, 2008 | In vivo and in vitro animal study | 2008 | Mouse | Epigallocatechin-3-gallate | 7 | 7 |
| Lebovic et al, 2007 | Prospective, randomized, placebo-controlled animal study | 2007 | Baboons | Rosiglitazone | 6 | 6 |
| Leconte et al, 2011 | In vivo and in vitro animal study | 2011 | Mice (C57BL/6 strain) | Temsirolimus | 20 | 20 |
| Onalan et al, 2013 | Prospective, randomized controlled animal study | 2013 | Rats | Amifostine, N-acetyl cysteine (NAC), and leuprolide acetate | 10 | 10 |
| Ozawa et al, 2006 | Prospective placebo-controlled study. | 2006 | Mouse | NS398 (Selective COX-2 inhibitor) | 23 Total | 23 Total |
| Ozer et al, 2013 | Prospective, randomized controlled animal study | 2012 | Mouse | Bevacizumab, Sorafenib, and Retinoic Acid | 10 | 10 |
| Peeters et al, 2005 | In vitro study | 2005 | Human endometrial cells (primary cultures) | Rosiglitazone (PPAR-γ agonists) | 80000 cells | 80000 cells |
| Rudzitis-Auth et al, 2013 | Prospective, randomized controlled animal study | 2013 | Mouse (BALB/c mice) | Resveratrol | 10 | 10 |
| Vlahos et al, 2010 | Prospective, randomized controlled animal study | 2010 | Mouse | Pentoxifylline | 10 | 10 |
| Wang et al, 2011 | In vitro study | 2011 | Human endometriotic stromal cells | Puerarin | Not specified | Not specified |
| Xu et al, 2011 | Prospective, randomized controlled animal study | 2011 | Mouse | Epigallocatechin-3-gallate | 10 | 10 |
| Xu H et al, 2012 | Prospective, randomized controlled animal study | 2012 | Mouse (BALB/c mice) | Lipoxin A4 (LXA4) | 20 | 20 |
| Zhang et al, 2011 | Prospective, randomized controlled animal study | 2011 | Rats (Female Sprague-Dawley rats) | Curcumin | 10 | 8 |

**Table S1.** Included study characteristics.

| Author, Year | Bias due to confounding | Bias in classification of interventions | Bias in selection of participants into the study (or into the analysis) | Bias due to deviations from intended interventions | Bias due to missing data | Bias in measurement of the outcome | Bias in selection of the reported result | Overall risk of bias |
| --- | --- | --- | --- | --- | --- | --- | --- | --- |
| Foda et al., 2012 | Low risk | Low risk | Moderate risk | Low risk | Moderate risk | Moderate risk | Moderate risk | Moderate risk |
| Filindris et al., 2024 | Moderate risk | Moderate risk | Low risk | Low risk | Moderate risk | Moderate risk | Moderate risk | Moderate risk |
| Khodarahmian et al., 2021 | Moderate risk | Moderate risk | Moderate risk | Moderate risk | Serious risk | Moderate risk | Moderate risk | Moderate risk |
| Moggio et al., 2012; | Moderate risk | Moderate risk | Moderate risk | Moderate risk | Low risk | Moderate risk | Moderate risk | Moderate risk |
| Okada et al., 2001 | Moderate risk | Moderate risk | Moderate risk | Moderate risk | Low risk | Moderate risk | Moderate risk | Moderate risk |
| Gomez et al., 2011 | Moderate risk | Moderate risk | Moderate risk | Moderate risk | Low risk | Moderate risk | Moderate risk | Moderate risk |

**Table S2.** ROBINS-I Risk of Bias assessment.

| Author, Year | Sequence generation | Baseline characteristics | Allocation concealment | Random housing | Blinding | Random outcome assessment | Blinding | Incomplete outcome data | Selective outcome reporting | Other sources of bias | Overall Score |
| --- | --- | --- | --- | --- | --- | --- | --- | --- | --- | --- | --- |
| Arablou et al., 2021 | Unclear | Yes | No | Unclear | No | No | No | No | No | No | Fair |
| Nap et al., 2004 | Unclear | Yes | No | Unclear | No | No | No | No | No | No | Fair |
| Machado et al., 2008 | Unclear | Yes | No | Unclear | No | No | No | No | No | No | Fair |
| Rein et al., 2010 | Unclear | No | No | Unclear | No | No | No | No | No | No | Poor |
| Santulli et al., 2015 | Unclear | No | No | Unclear | No | No | No | No | No | No | Poor |
| Huang et al., 2013 | Unclear | No | No | Unclear | No | No | No | No | No | No | Poor |
| Abbas et al, 2013 | Unclear | No | No | Unclear | No | No | No | No | No | No | Poor |
| Edwards et al, 2014 | Unclear | No | No | Unclear | No | No | No | No | No | No | Poor |
| Hussein et al, 2016 | Unclear | No | No | Unclear | No | No | No | No | No | No | Poor |
| Laschke et al, 2006 | Unclear | Yes | No | Unclear | No | No | No | No | No | No | Fair |
| Laschke et al, 2008 | Unclear | Yes | No | Unclear | No | No | No | No | No | No | Poor |
| Lebovic et al, 2007 | Unclear | No | No | Unclear | No | No | No | No | No | No | Fair |
| Leconte et al, 2011 | Unclear | Yes | No | Unclear | No | No | No | No | No | No | Fair |
| Onalan et al, 2013 | Unclear | Yes | No | Unclear | No | No | No | No | No | No | Fair |
| Ozawa et al, 2006 | Unclear | Yes | No | Unclear | No | No | No | No | No | No | Fair |
| Ozer et al, 2013 | Unclear | Yes | No | Unclear | No | No | No | No | No | No | Fair |
| Rudzitis-Auth et al, 2013 | Unclear | Yes | No | Unclear | No | No | No | No | No | No | Fair |
| Vlahos et al, 2010 | Unclear | No | No | Unclear | No | No | No | No | No | No | Poor |
| Xu et al, 2011 | Unclear | Yes | No | Unclear | No | No | No | No | No | No | Fair |
| Xu H et al, 2012 | Unclear | Yes | No | Unclear | No | No | No | No | No | No | Fair |
| Zhang et al, 2011 | Unclear | Yes | No | Unclear | No | No | No | No | No | No | Fair |

**Table S3.** SYRCLE Risk of bias assessment

| Outcome | n_contrasts | Egger_intercept | Egger_p_value | Interpretation |
| --- | --- | --- | --- | --- |
| Lesion number | 11 | -3.303708523 | 0.28516715 | No strong evidence of small-study effects (p >= 0.10) |
| Lesion size | 33 | -4.377811171 | 0.003309882 | Possible small-study effects (Egger p < 0.10) |
| VEGF expression | 16 | -8.520417756 | 3.40E-05 | Possible small-study effects (Egger p < 0.10) |
| MVD | 9 | -5.384585607 | 0.008607496 | Possible small-study effects (Egger p < 0.10) |
| Apoptosis | 4 | 17.28712918 | 0.077686044 | Possible small-study effects (Egger p < 0.10) |
| Proliferation | 7 | -2.35168986 | 0.802412816 | No strong evidence of small-study effects (p >= 0.10) |

**Table S4**. Eggers test summary for asymmetry per outcome.

| **Database** | **Pathway (official term)** | **Genes in list / pathway size** | **Enrichment (%)** | **Hyper-geometric P-value†** |
| --- | --- | --- | --- | --- |
| **KEGG** | Apoptosis (hsa04210) | 11 / 140 | **7.9 %** | 1.2 × 10⁻¹⁵ |
|  | PI3K-Akt signalling (hsa04151) | 11 / 350 | 3.1 % | 4.6 × 10⁻¹² |
|  | MAPK signalling (hsa04010) | 8 / 266 | 3.0 % | 7.8 × 10⁻¹⁰ |
|  | p53 signalling (hsa04115) | 5 / 73 | 6.8 % | 2.1 × 10⁻⁸ |
|  | VEGF signalling (hsa04370) | 5 / 60 | 8.3 % | 4.9 × 10⁻⁸ |
|  | Cytokine–cytokine receptor interaction (hsa04060) | 4 / 260 | 1.5 % | 6.2 × 10⁻⁵ |
| **Reactome** | Programmed cell death (R-R-HSA-5357801) | 12 / 300 | **4.0 %** | 3.4 × 10⁻¹³ |
|  | Intrinsic caspase cascade (R-R-HSA-6803205) | 7 / 92 | 7.6 % | 1.9 × 10⁻¹¹ |
|  | RAF/MAP kinase cascade (R-R-HSA-5673001) | 7 / 165 | 4.2 % | 6.0 × 10⁻¹⁰ |
|  | PI3K/AKT activation (R-R-HSA-450347) | 7 / 180 | 3.9 % | 1.1 × 10⁻⁹ |
|  | VEGF ligand–receptor interactions (R-R-HSA-2980584) | 4 / 58 | 6.9 % | 8.5 × 10⁻⁷ |
|  | TP53-regulated transcription (R-R-HSA-5633007) | 4 / 70 | 5.7 % | 2.6 × 10⁻⁶ |

| **Gene** | **n** |
| --- | --- |
| ABCB11 | 2 |
| AKT1 | 2 |
| BAK1 | 2 |
| BAX | 2 |
| BCL2L1 | 2 |
| CASP3 | 4 |
| CASP7 | 3 |
| CASP8 | 3 |
| CASP9 | 2 |
| CAT | 2 |
| CDKN1A | 2 |
| CYP1A1 | 4 |
| CYP2C9 | 2 |
| EGF | 2 |
| ESR1 | 2 |
| F3 | 2 |
| FAS | 2 |
| MAPK1 | 3 |
| MAPK3 | 3 |
| MAPK8 | 3 |
| MAPK14 | 2 |
| MMP2 | 2 |
| MMP9 | 2 |
| MTOR | 3 |
| NR1H4 | 2 |
| NR1I2 | 2 |
| PPARG | 2 |
| PTGS2 | 2 |
| RET | 2 |
| SIRT3 | 2 |
| SOD2 | 2 |
| SP1 | 3 |
| SRC | 2 |
| STAT3 | 2 |
| TGFB1 | 2 |
| TNFSF10 | 2 |
| TP53 | 3 |

**Table S5.** Genes present in at least two drugs (N = 37) were interrogated for functional over-representation against KEGG Reactome via cluster Profiler 4.10 and cross-validated in g:Profiler2 (Raudvere et al., 2019), adopting a Benjamini–Hochberg FDR < 0.05.

| Drug pair | Overlap ( n genes ) | Shared gene symbols |
| --- | --- | --- |
| **Retinoic Acid + Sorafenib** | **11** | CASP3, CASP7, CASP8, CASP9, CYP2C9, FAS, MAPK1, MAPK3, **RET**, TGFB1, TNFSF10 |
| **EGCG (E3G) + Retinoic Acid** | **10** | **EGF**, F3, FOS, MAPK1, MAPK14, MAPK3, NR1H4, SOD2, **SP1**, **TP53** |
| **Resveratrol + Retinoic Acid** | **6** | **AKT1**, **CDKN1A**, **NCOA3**, **PPARG**, SP1, TP53 |
| **Metformin + Retinoic Acid** | **5** | CASP3, CASP7, CASP8, MMP2, NR1I2 |
| **EGCG + Sorafenib** | **4** | CYP1A1, MAPK1, MAPK3, **STAT3** |
| **Metformin + Sorafenib** | **4** | CASP3, CASP7, CASP8, CYP1A1 |
| **Resveratrol + EGCG** | **3** | MAPK8, SP1, TP53 |
| **EGCG + Metformin** | **3** | **CAT**, CYP1A1, **MMP9** |
| **Resveratrol + Metformin** | **2** | **ESR1**, **SIRT3** |
| **Resveratrol + Sorafenib** | **2** | **MTOR**, **SRC** |
| **GnRH agonist + Sorafenib** | **2** | **ABCB11**, CASP3 |
| **GnRH agonist + Metformin** | **1** | CASP3 |
| **GnRH agonist + Retinoic Acid** | **1** | CASP3 |
| **Parecoxib + Resveratrol** | **1** | **PTGS2** |
| **All other pairs** (including any with Bevacizumab) | 0 | — |

**Table S6.** Overlap of drug-gene (N=16) intreractome as depicted in Figure 3.

| **Fertility-sparing candidates** | **Pathway-coverage** | **Fertility-non-sparing candidates** | **Pathway-coverage** |
| --- | --- | --- | --- |
| Curcumin (nutraceutical; GRAS) | **14 / 16 = 87 %** |  |  |
| Resveratrol (nutraceutical) | 6 / 16 = 38 % | Sorafenib (multi-kinase) | 6 / 16 = 38 % |
| Epigallocatechin-3-gallate | 5 / 16 = 31 % | Sirolimus / everolimus (mTOR) | 5 / 16 = 31 % |
| Metformin | 4 / 16 = 25 % | Bentamapimod (JNK) | 3 / 16 = 19 % |
| Tacrolimus (calcineurin) | 3 / 16 = 19 % | GnRH antagonists (ganirelix / cetrorelix) | 2 / 16 = 13 % |
| Dopamine agonists (quinagolide / cabergoline) | 1 / 16 = 6 % |  |  |

**Table S7.** Rapid pathway-coverage screen of human-approved drugs. Using the 16 “hall-mark” genes that drove KEGG/Reactome enrichment (CASP3/7/8/9, BAX, BAK1, BCL2L1, FAS, TNFSF10, MAPK1/3/14, STAT3, TP53, CDKN1A, SIRT3) we over-laid the curated target lists of readily available medications (Drug Bank + STITCH confidence ≥ 0.4). Coverage is expressed as % of these 16 genes hit at least once by the drug.
