# Supplementary material for: Targeting angiogenesis in endometriosis: a systematic review and network meta-analysis of VEGF-directed pharmacotherapies
Source: Front Reprod Health. 2026 Jan 26;7:1744465. doi: 10.3389/frph.2025.1744465 (PMC12883779; doi:10.3389/frph.2025.1744465)
Supplement: Supplementary file 8 [file Supplementaryfile2.docx]

Title. Targeting Angiogenesis in Endometriosis: A Systematic Review and Network Meta-analysis of VEGF-Directed Pharmacotherapies.

**Authors** : Hammond O.1, El-Sheikh O.1, Saad RM.2, Shetty P.1, Papakonstantinou E.3 Kastora SL.4


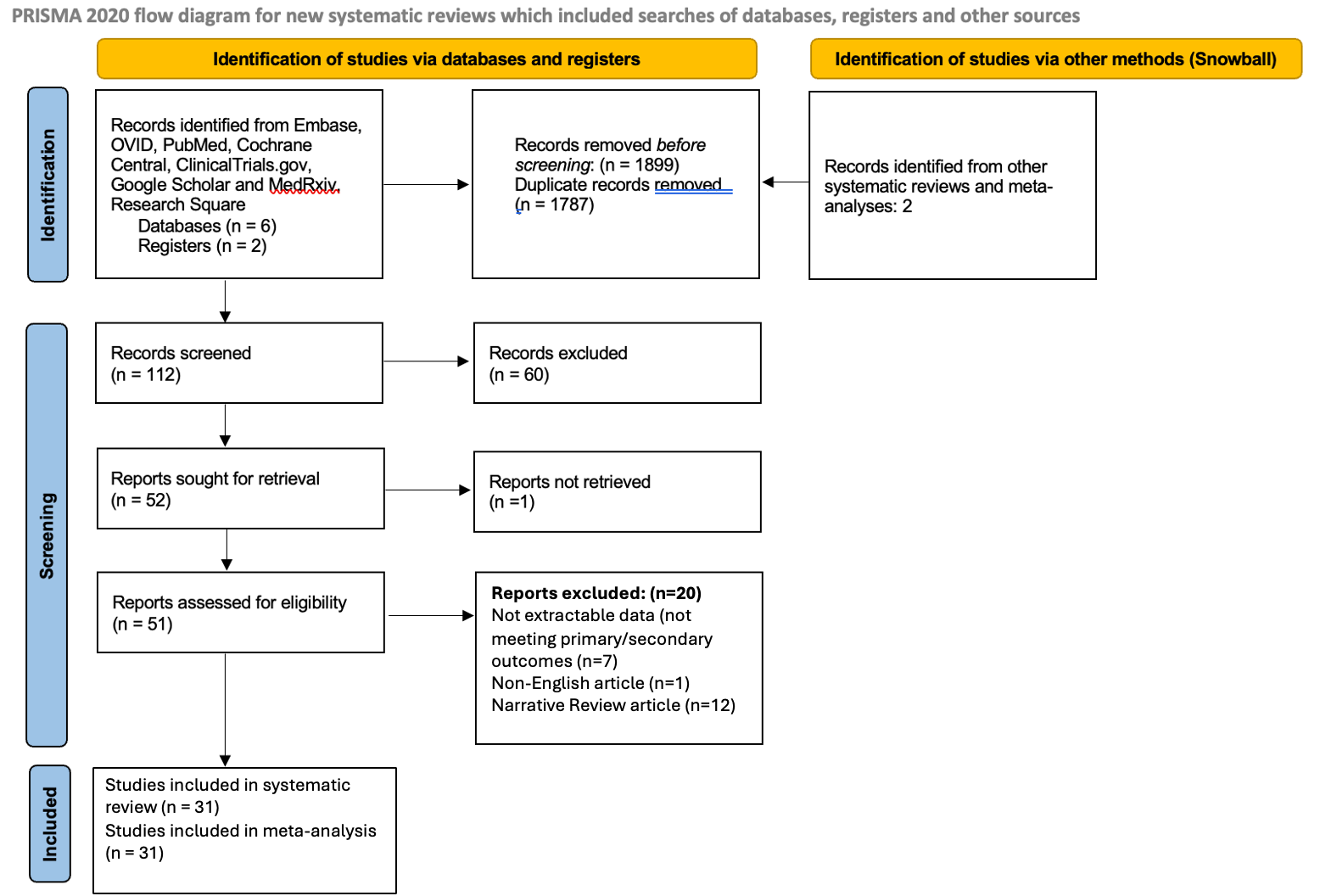


Figure S1. PRISMA Chart.


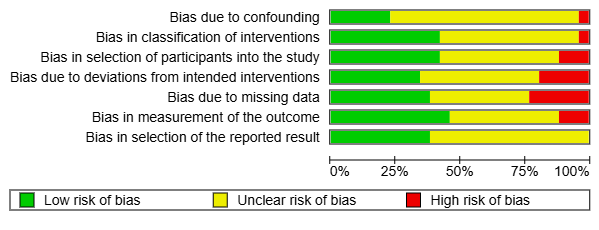


Figure S2. Risk of Bias assessment of included studies.


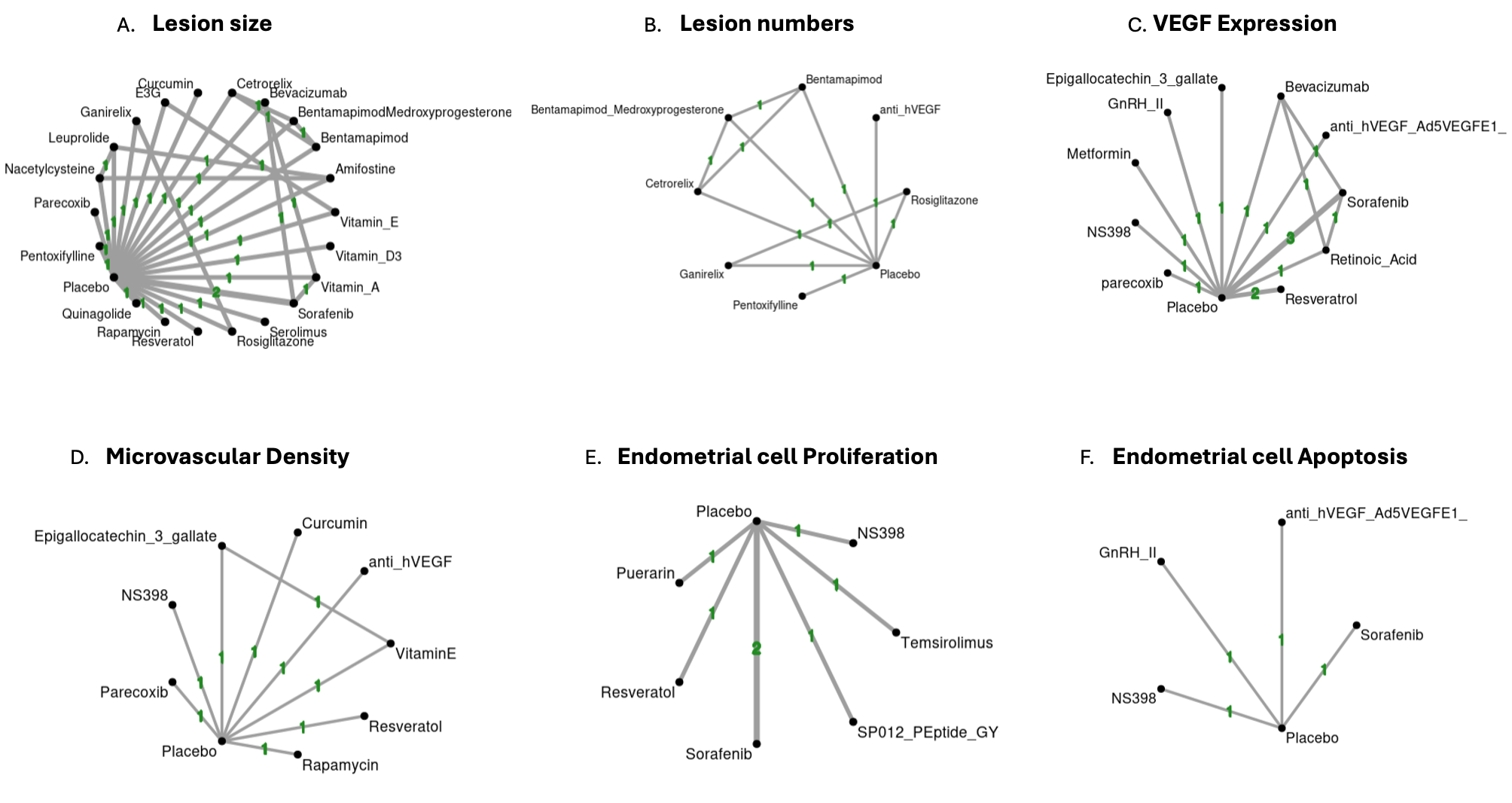


Figure S3. Networks per outcome.


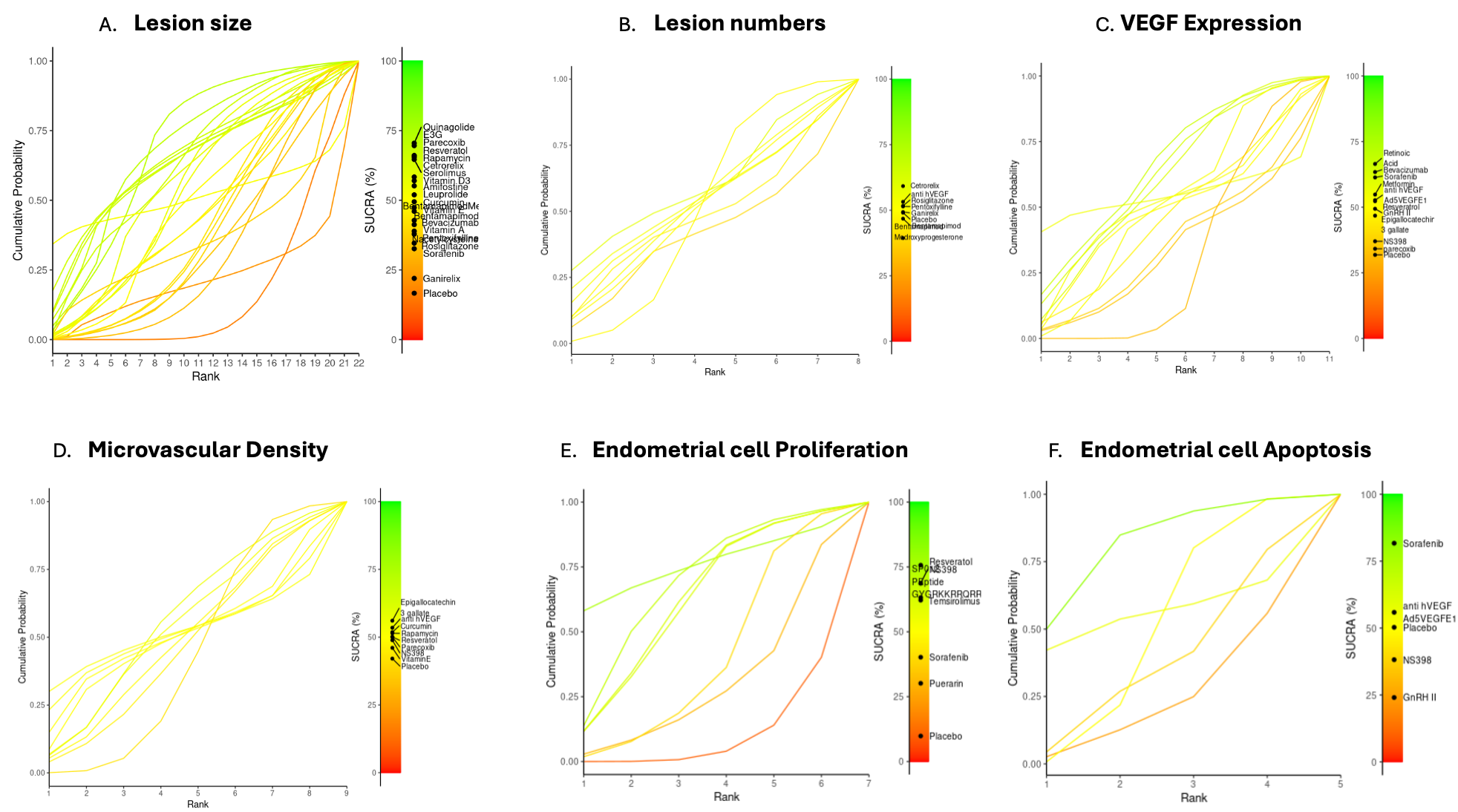


Figure S4. SUCRA Ranking plots.


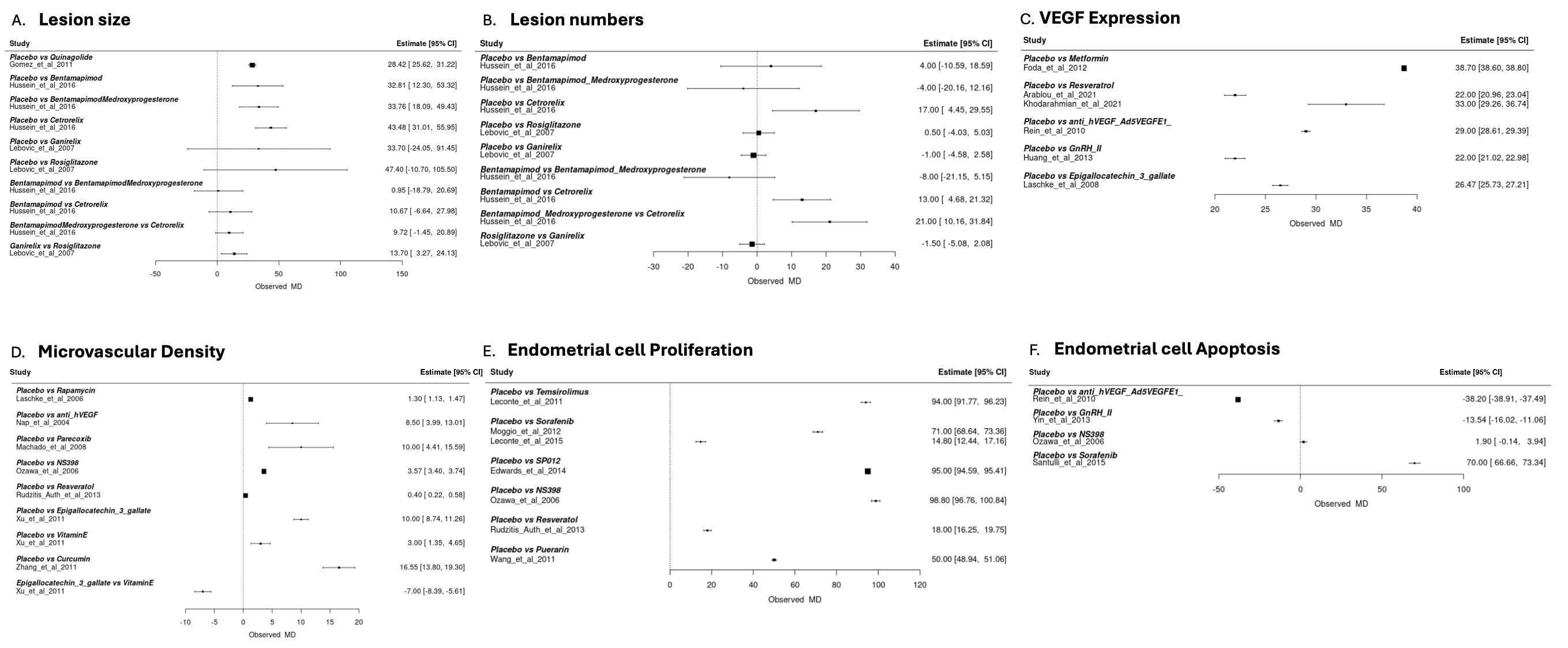


Figure S5. Individual study results (all included studies) grouped by treatment comparison.
